# Supplementary material for: Effectiveness of Physiotherapy in Patients with Ankylosing Spondylitis: A Systematic Review and Meta-Analysis
Source: Healthcare (Basel). 2022 Jan 10;10(1):132. doi: 10.3390/healthcare10010132 (PMC8775656; doi:10.3390/healthcare10010132)
Supplement: Supplementary file 1 [file healthcare-10-00132-s001.zip › healthcare-1524812-supplementary.pdf]

Summary of findings:

Interventions compared to Usual care in patients with Ankylosis Spondylitis

Patient or population: patients with Ankylosis Spondylitis
Setting: rehabilitation setting
Intervention: Interventions
Comparison: Usual care

Outcomes	Anticipated absolute effects* (95% CI)		Relative effect (95% CI)	№ of participants (studies)	Certainty of the evidence (GRADE)	Comments
	Risk with Usual care	Risk with Interventions				
Functional Index (BASFI) assessed with: Bath Ankylosing Spondylitis Functional Index follow-up: median 12 weeks	-	SMD **0.34 SD lower** (0.53 lower to 0.14 lower)	-	1247 (8 RCTs)	⨁◯◯◯ Very low[a,b,c,d]	Interventions may reduce functional Index but the evidence is very uncertain.
Activity disease (BASDAI) assessed with: Bath Ankylosing Spondylitis Disease Activity Index Scale from: 0 to 10 follow-up: median 12 weeks	-	SMD **0.37 SD lower** (0.64 lower to 0.11 lower)	-	1247 (8 RCTs)	⨁◯◯◯ Very low[a,b,c,d]	Interventions may reduce activity disease but the evidence is very uncertain.
Metrology (BASMI) assessed with: Bath Ankylosing Spondilytis Metrology Index Scale from: 0 to 10 follow-up: median 12 weeks	-	SMD **0.12 SD lower** (0.33 lower to 0.08 higher)	-	362 (5 RCTs)	⨁⨁◯◯ Low[a,d]	The evidence suggests interventions reduces metrology slightly.
Pain (VAS) assessed with: Visual Analogue Scale Scale from: 0 to 100 follow-up: mean 16 weeks	-	SMD **0.31 SD lower** (0.88 lower to 0.25 higher)	-	813 (2 RCTs)	⨁◯◯◯ Very low[c,d,e]	The evidence is very uncertain about the effect of interventions on pain.
Quality of Life (ASQoL) assessed with: Ankylosing Spondylitis Quality of Life Scale from: 0 to 18 follow-up: mean 16 weeks	-	SMD **0.09 SD lower** (0.51 lower to 0.32 higher)	-	866 (3 RCTs)	⨁◯◯◯ Very low[a,b,d]	The evidence is very uncertain about the effect of interventions on quality of Life.

***The risk in the intervention group** (and its 95% confidence interval) is based on the assumed risk in the comparison group and the **relative effect** of the intervention (and its 95% CI).

CI: confidence interval; **SMD:** standardised mean difference

GRADE Working Group grades of evidence
High certainty: we are very confident that the true effect lies close to that of the estimate of the effect.
Moderate certainty: we are moderately confident in the effect estimate: the true effect is likely to be close to the estimate of the effect, but there is a possibility that it is substantially different.
Low certainty: our confidence in the effect estimate is limited: the true effect may be substantially different from the estimate of the effect.
Very low certainty: we have very little confidence in the effect estimate: the true effect is likely to be substantially different from the estimate of effect.

Explanations

a. Any studies with low risk of bias
b. No direct comparison between the interventions
c. Etherogenety >60%
d. Different and few follow-up
e. All studies with high risk of bias

Summary of findings:

Intervention compared to Home-based exercise programs for Patients with ankylosing spondylitis

Patient or population: Patients with ankylosing spondylitis
Setting: Rehabilitation clinic, home
Intervention: Intervention
Comparison: Home-based exercise programs

Outcomes	Anticipated absolute effects* (95% CI)		Relative effect (95% CI)	№ of participants (studies)	Certainty of the evidence (GRADE)	Comments
	**Risk with Home-based exercise programs**	**Risk with Intervention**				
Functional Index (BASFI) assessed with: Bath Ankylosing Spondylitis Functional Index Scale from: 0 to 10 follow-up: median 12 weeks	-	SMD **0.29 SD lower** (0.79 lower to 0.12 higher)	-	236 (4 RCTs)	⨁⨁◯◯ Low[a,b]	The evidence suggests intervention results in a slight reduction in functional Index.
Activity disease (BASDAI) assessed with: Bath Ankylosing Spondylitis Disease Activity Index Scale from: 0 to 10 follow-up: median 12 weeks	-	SMD **0.14 SD lower** (0.42 lower to 0.15 higher)	-	191 (3 RCTs)	⨁⨁◯◯ Low[c,d]	The evidence suggests intervention results in a slight reduction in activity disease.
Metrology (BASMI) assessed with: Bath Ankylosis Arthritis Metrology Index Scale from: 0 to 10 follow-up: median 12 weeks	-	SMD **0.2 SD lower** (0.77 lower to 0.37 higher)	-	191 (3 RCTs)	⨁⨁⨁◯ Moderate[d]	Intervention probably results in a reduction in metrology.
Pain (VAS) assessed with: Visual Analogue Scale Scale from: 0 to 100 follow-up: median 12 weeks	-	SMD **0.27 SD lower** (0.61 lower to 0.07 higher)	-	166 (3 RCTs)	⨁⨁⨁◯ Moderate[d]	Intervention likely results in a slight reduction in pain.
Quality of life (ASQoL) assessed with: Ankylosing Spondylitis Quality of Life follow-up: mean 4 weeks	-	SMD **0.75 SD lower** (1.31 lower to 0.2 lower)	-	52 (1 RCT)	⨁⨁◯◯ Low[d,e]	The evidence suggests that intervention results in little to no difference in quality of life.

***The risk in the intervention group** (and its 95% confidence interval) is based on the assumed risk in the comparison group and the **relative effect** of the intervention (and its 95% CI).

CI: confidence interval; **SMD:** standardised mean difference

Summary of findings:

Intervention compared to Home-based exercise programs for Patients with ankylosing spondylitis

Patient or population: Patients with ankylosing spondylitis
Setting: Rehabilitation clinic, home
Intervention: Intervention
Comparison: Home-based exercise programs

Outcomes	Anticipated absolute effects* (95% CI)		Relative effect (95% CI)	№ of participants (studies)	Certainty of the evidence (GRADE)	Comments
	Risk with Home-based exercise programs	Risk with Intervention				

GRADE Working Group grades of evidence
High certainty: we are very confident that the true effect lies close to that of the estimate of the effect.
Moderate certainty: we are moderately confident in the effect estimate: the true effect is likely to be close to the estimate of the effect, but there is a possibility that it is substantially different.
Low certainty: our confidence in the effect estimate is limited: the true effect may be substantially different from the estimate of the effect.
Very low certainty: we have very little confidence in the effect estimate: the true effect is likely to be substantially different from the estimate of effect.
